# Association Analysis of TP53 rs1042522, MDM2 rs2279744, rs3730485, MDM4 rs4245739 Variants and Acute Myeloid Leukemia Susceptibility, Risk Stratification Scores, and Clinical Features: An Exploratory Study

**DOI:** 10.3390/jcm9061672

**Published:** 2020-06-01

**Authors:** Florin Tripon, Mihaela Iancu, Adrian Trifa, George Andrei Crauciuc, Alina Boglis, Beata Balla, Adriana Cosma, Delia Dima, Marcela Candea, Erzsebet Lazar, Laura Jimbu, Claudia Banescu

**Affiliations:** 1 Genetics Department, George Emil Palade University of Medicine, Pharmacy, Science, and Technology of Targu Mures, 540139 Targu Mures, Romania; tripon.florin.2010@gmail.com (F.T.); andrei.crauciuc@gmail.com (G.A.C.); alinaboglis@gmail.com (A.B.); beakardos@gmail.com (B.B.); adriana.cosma17@gmail.com (A.C.); claudia.banescu@gmail.com (C.B.); 2 Genetics Laboratory, Center for Advanced Medical and Pharmaceutical Research of George Emil Palade University of Medicine, Pharmacy, Science, and Technology of Targu Mures, 540139 Targu Mures, Romania; 3 Department of Medical Informatics and Biostatistics, “Iuliu Hatieganu” University of Medicine and Pharmacy, 400000 Cluj Napoca, Romania; 4 Department of Medical Genetics, “Iuliu Hatieganu” University of Medicine and Pharmacy, 400000 Cluj-Napoca, Romania; 5 Department of Hematology, “Ion Chiricuta” Clinical Cancer Center, 400015 Cluj-Napoca, Romania; deli_dima@yahoo.com (D.D.); jimbulaura@yahoo.com (L.J.); 6 Department of Internal Medicine, George Emil Palade University of Medicine, Pharmacy, Science, and Technology of Targu Mures, 540139 Targu Mures, Romania; marcela1212ro@yahoo.com (M.C.); erzsebetlazarbenedek@gmail.com (E.L.); 7 Department of Hematology, “Iuliu Hatieganu” University of Medicine and Pharmacy, 400000 Cluj-Napoca, Romania

**Keywords:** acute myeloid leukemia, rs1042522 (*TP53* Arg72Pro), rs2279744 (*MDM2* 309T>G), rs3730485 (*MDM2* del1518), rs4245739 (*MDM4* 34091 C>A)

## Abstract

This study aimed to explore the associations between the *TP53* rs1042522 (*TP53* Arg72Pro), *MDM2* rs2279744 *(MDM2* 309T>G), rs3730485 *(MDM2* del1518), *MDM4* rs4245739 (*MDM4* 34091 C>A) variants and odds of developing acute myeloid leukemia (AML) in a cohort of 809 adult subjects, consisting of 406 healthy controls and 403 AML patients. Model-based multifactor dimensionality reduction (MB-MDR) framework was used to identify the interactions of the mentioned variants and their association with AML risk. Associations of the mentioned variants with clinical features of AML, somatic mutations, and response to treatment were also evaluated. Significant associations between *TP53* rs1042522 and *MDM4* rs4245739 variants and AML susceptibility were noticed. MB-MDR and logistic regression analysis revealed an interaction between *MDM2* rs2279744 and *TP53* rs1042522, between *MDM4* rs4245739 and *MDM2* rs3730485, as well as significant associations with AML susceptibility. Several associations between the mentioned variants and clinical features of AML and somatic mutations were also noticed. Individually, the variant genotypes of *TP53* rs1042522 and *MDM4* rs4245739 were associated with AML susceptibility, but their interaction with *MDM2* rs2279744 and rs3730485 modulated the risk for AML. The variant genotypes of *TP53* rs1042522 were associated with adverse molecular and cytogenetic risk and also with *NPM1* mutations.

## 1. Introduction

Acute myeloid leukemia (AML) patients may have one or several genetic abnormalities. More than 90% of the AML patients had genetic abnormalities. Frequently, the genetic alteration involved *Tumor protein p53 (TP53)* gene. This gene has an important role in apoptosis and DNA-damage response [1]. *TP53* gene encodes the p53 protein. *TP53* gene expression is inhibited by *Mouse double minute 2 homolog (MDM2)* gene and by *MDM4* gene (homolog of *MDM2*) [2,3], *MDM2*, and *MDM4* genes being negative regulators of p53 [4].

Previously it was reported that approximately 30% of acute lymphoid leukemia (ALL) patients and 47% of AML patients had over-expressed the MDM2 protein compared to the control group (at least 10-fold). Over-expression of MDM2 protein being correlated for AML patients with a worse prognostic [1,5] *MDM4* gene is the homolog of the *MDM2* gene and their codified proteins showed very high structural and functional similarities, both inhibiting p53 protein by complex mechanisms described by Qin L et al. [6]. Therefore, the inhibition of p53 by over-expression of MDM2 and MDM4 thereby can contribute to leukemogenesis.

In malignancies, including leukemia, *TP53* gene expression was reported to be affected by a missense variant, rs1042522 (*TP53* Arg72Pro) [7,8]. Moreover, the variant allele of rs1042522 was reported to decrease apoptotic activity, increase the risk of cancer (including leukemia), and to be associated with poor overall survival (OS) in patients with AML and higher rate of refractory disease [2,7,9,10,11].

However, the role of rs1042522 in AML susceptibility and OS is not fully clarified due to different results reported in the literature. Six studies performed on AML patients were included in a recent meta-analysis [7] including 200 Japanese patients [12], 171 patients from UK and USA [13], 411 Chinese patients from two studies (231+180) [1,14], and 272 Indian patients from two studies (131+141) [15,16]. While no association was found between AML risk and variant allele of rs1042522, different associations with OS and response to treatment were noticed. On the other hand, one recent study performed on 189 Brazilian AML patients described an association between rs1042522 and AML risk [11]. One cause of contradictory results may be the small cohorts of the mentioned studies and different ethnicity of the subjects [2,11].

Similarly, it was reported that expression of *MDM2* and *MDM4* genes are affected by *MDM2* rs2279744 (*MDM2* 309T>G) and rs3730485 (*MDM2* del1518) respectively by *MDM4* rs4245739 (*MDM4* 34091 C>A) variants, the mentioned variants being associated with increased risk for different types of cancer and higher incidence of advanced tumor stages [17,18,19,20]. In contradiction with the abovementioned results, Gansmo LB et al. [21] in their study among endometrial cancer patients found that rs3730485 was in strong linkage disequilibrium (LD) with rs2279744 and the variant allele of rs3730485 was associated with reduced cancer risk among patients with the wildtype genotype for rs2279744 [21]. Lian T et al. [22] in their meta-analysis suggested that variant allele of rs4245739 was associated with reduced cancer risk, especially in Asian populations [22]. Another meta-analysis performed by Hua W et al. [23] on Asian and Caucasian populations reported no associations between rs3730485 variant and cancer risk. However, none of the mentioned meta-analyses included AML patients. 

Regarding the risk for AML, Soleymannejad M et al. [24] noticed an association between *MDM2* rs2279744 and AML susceptibility. Also, Phillips CL et al. [25] reported the same association on childhood AML patients and no association with treatment response [25]. Moreover, Falk IJ et al. [26] showed association with lower OS in adult AML patients. On the other hand, Abdel TM et al. [27] did not find an association between rs2279744 and lower OS. In other hematological malignancies, such as chronic lymphoid leukemia, the variant genotype of rs2279744 was reported to be associated with a nine-fold increase in the risk of death [28]. Moreover, the simultaneous presence of wildtype allele of *TP53* rs1042522 and the variant allele of *MDM2* rs2279744 may influence the risk of therapy-related myeloid neoplasms [29]. *MDM2* rs3730485 and *MDM4* rs4245739 variants were studied in different types of cancer, together or in combination with one or with the other two mentioned variants, but from our knowledge, AML patients were not investigated. None of the published studies investigated the *TP53* rs1042522, *MDM2* rs2279744, rs3730485, and *MDM4* rs4245739 variants simultaneously. Moreover, none of the published studies focused on identifying the interactions of the mentioned variants and their association with AML (or other cancers) risk.

First of all, our study aimed to explore the associations between the *TP53* rs1042522 (*TP53* Arg72Pro), *MDM2* rs2279744 (*MDM2* 309T>G), rs3730485 (*MDM2* del1518), and *MDM4* rs4245739 (*MDM4* 34091 C>A) variants and odds of developing AML in a cohort of 809 adult subjects, consisting of 406 healthy controls and 403 AML patients. In addition, we analyzed the pairwise and higher-order interactions of investigated *TP53*, *MDM2,* and *MDM4* variants and their association with odds of AML. Moreover, we evaluated the associations of the mentioned variants with the clinical features of AML, somatic mutations, and response to treatment.

## 2. Materials and Methods

### 2.1. Patients and Controls

The Board of the Ethical Committee of the Clinical and Emergency Hospital of Targu Mures, Romania, approved this case-control study (10665/2019). The subjects included signed a written informed consent form. The study was performed in accordance with the fundamental principles of the Declaration of Helsinki. A total number of 809 adult subjects were included, 406 healthy controls and 403 AML patients. AML group consisted of 215 males and 188 females while the control group was comprised of 181 males and 225 females. We included only subjects who signed the written informed consent form, with complete laboratory and clinical records, in which the genotyping investigation was successfully performed. AML diagnosis was based on clinical examination and laboratory investigation (including complete blood count, blood smear, bone marrow and/or blood microscopic examination, flow cytometry, cytogenetics, fusion gene investigation as reported previously [30], DNA copy number variations analysis as reported previously [31], fragment analysis for FLT3-ITD and NPM1 mutations as reported previously [32,33,34], and target next-generation sequencing as reported previously [34]).

### 2.2. Genotyping Investigation

For DNA isolation, we used the manufacturer’s protocol of PureLink Genomic DNA kit (Thermo Fisher Scientific, Carlsbad, CA, USA) or Quick-DNA Miniprep Plus Kit (ZymoResearch, Irvine, CA, USA). Total DNA was purified from nucleated blood cells. Genotyping protocols used for TP53 rs1042522, MDM2 rs3730485, and MDM4 rs4245739 variants were previously published [21,35,36,37,38]. The protocols were in-silico checked using the following free tools: Primer-BLAST (National Center for Biotechnology Information, NCBI), in-silico PCR program (University of California, Santa Cruz, UCSC In-Silico PCR—UCSC Genome Browser), NEBcutter V2.0 for RFLP-PCR technique (New England BioLabs, Ipswich, MA, USA) and SNPCheck 3 program (National Genetics Reference Laboratories, NGRL Manchester). For MDM2 rs2279744 genotyping we used TaqMan assay (C__15968533_20, Thermo Fisher Scientific, Carlsbad, CA, USA) and 7500 Fast Dx Real-Time PCR system (Applied Biosystems, Foster City, CA, USA). The genotypes of 10% of the subjects were confirmed by capillary sequencing (3500 Genetic Analyzer, Applied Biosystems, Foster City, CA, USA).

NCBI’s (NCBI Homo sapiens Annotation Release 109), Ensembl’s (Ensembl Release 98) and European Variation Archive’s genome browser were used for alleles annotation of the investigated variants. 

### 2.3. Statistical Analysis

#### 2.3.1. Descriptive Analysis

The demographic, clinical, and genetic data were presented via descriptive measures as mean ± standard deviation or percentages and absolute frequencies. 

#### 2.3.2. Inferential Analysis

The Hardy–Weinberg equilibrium (HWE) and pairwise linkage disequilibrium (LD), using the standardized D, were performed both in AML and control group.

The statistical associations of *TP53* rs1042522, *MDM2* rs2279744, rs3730485, and *MDM4* rs4245739 variants with demographic, clinical features, and somatic mutations were assessed using Chi-square/Fisher’s Exact test. Post-hoc pairwise Chi-square or Fisher tests were performed after significant association have been identified and adjusted *p*-values computed via Benjamini–Hochberg procedure were reported.

The associations between selected genetic variants and odds of AML were estimated in different genetic models: Codominant, dominant, recessive, and overdominant using generalized linear models (GLMs) with a binomial distribution (logit as link function). In addition, the univariate effect of selected variants on odds of AML was adjusted for age group (≥60 years) and gender. The Benjamini–Hochberg procedure based on false discovery rate (FDR) criterion was performed in order to adjust for multiple genetic comparisons.

The MB-MDR method was performed to identify the potential higher-order gene–gene interactions among the selected genetic variants [39]. The *mbmdr* package (version 2.6) for R (version 3.6.1) obtained from the CRAN repository was used to apply the MB-MDR method [40]. For all studied genetic variants, codominant genetic model was assumed and the second, third, and four-order variants combinations were investigated.

We used the default settings of mbmdr R package, and the genotypes combinations having the beta coefficients > 0 and *p*-value smaller than 0.10 were assigned to the “high-risk” group while the genotype combinations with the beta coefficients < 0 and *p*-value smaller than 0.10 were assigned to the “low-risk” group. In the case when *p*-value ≥ 0.10, genotype combinations were assigned to the “no-evidence” group. In the next step of MB-MDR analysis, the multi-locus genotypes with the same risk category were merged and a new variable with three categories (H = high risk, L = low risk, 0 = no-evidence) was created. The H and L risk groups were tested for association with odds of AML versus the remaining two groups by logistic regression with adjustment to main effects of single nucleotide polymorphisms (SNPs), age, and gender. The results were expressed by the beta regression coefficients for each risk category: β_H_ for high-risk group and β_L_ for high-risk group. Significance of the regression coefficients was evaluated by Wald statistics (W_H_ and W_L_) adjusted for the number of genotype combinations included in each risk category. Based on W_H_ and W_L_, *p*-values were calculated (p_H_ and p_L_) and the minimum of these two values was considered as the result of statistics test for the studied interaction effect. In order to adjust for multiple testing, the statistics (defined as the maximum between W_H_ and W_L_) was compared with the permutational distribution of the Wald statistic, using 1000 permutations, and the *p*-values obtained by permutation test were considered as corrected *p*-values.

All statistical tests were two-sided and significant at an estimated significance level *p* <0.05. All statistical analysis of data was performed in R software, version 3.6.1. 

## 3. Results

### 3.1. Description of AML and Control Groups

The mean ± standard deviation of age at diagnosis in AML cases was 56.51 ± 16.10 years and 56.85 ± 15.60 for control group. There was no statistically significant difference in overall mean age between the two groups (*p* = 0.760). In the present study, the frequency distribution of age-group categories of AML cases was found to be 48.64% (196 cases) in the ≥60 years age group while for the control group, 54.43% (221 controls) were classified in the ≥60 years age group (*p* = 0.106).

Regarding gender distribution, there was a significant difference in sex distribution between these two groups (*p* = 0.013), male individuals being more frequent in the AML group than in the control group (215, 53.34% versus 181, 44.58%) with a male-to-female ratio of 1.14 for AML group.

Regarding AML types, 316 (78.41%) of our patients were with de novo AML, 82 (20.35%) with secondary AML (sAML) and 5 (1.24%) developed therapy related AML. According to cytogenetic risk and European Leukemia Net (ELN) 2017 risk stratification scores, 81 (20.1%) and 105 (26.05%) AML patients were low risk, 223 (55.33%) and 183 (45.41%) were intermediate risk, and 89 (22.08%) and 78 (19.35%) were high risk, respectively.

### 3.2. TP53 rs1042522, MDM2 rs2279744, rs3730485, and MDM4 rs4245739 Variants and Odds of AML

Distribution of *TP53* rs1042522, *MDM2* rs2279744, rs3730485, and *MDM4* rs4245739 variants were tested for Hardy–Weinberg equilibrium (HWE) and no significant differences in the genotype frequencies were found except for *TP53* rs1042522 variant (*p* < 0.001) in the AML group and the *MDM4* rs4245739 variant in the two groups (*p* = 0.0004 for AML cases and *p* < 0.001 for controls).

The linkage disequilibrium (LD) analysis was performed both in AML cases and controls and there was a strong LD in controls (D’ = 0.89, *p* < 0.001) and AML group (D’ = 0.80, *p* < 0.001).

Distribution of the investigated variant genotypes is illustrated in Table 1. Four genetic models (codominant, dominant, recessive, and overdominant) were used to compare the distribution of genotypes between groups. The variant genotypes of *TP53* rs1042522 and *MDM4* rs4245739 were associated with odds of AML and they also remained significant predictors of AML risk after adjusting for gender and age-group.

### 3.3. TP53 rs1042522, MDM2 rs2279744, rs3730485, and MDM4 rs4245739 Interactions and Odds of AML

The results of epistatic pairwise interactions between studied variants are illustrated in Table 2. In recessive model, there was a significant effect of interaction between *MDM2* rs2279744 and *TP53* rs1042522 variants (*p* = 0.044 < 0.05) and between *MDM4* rs4245739 and *MDM2* rs3730485 variants (*p* = 0.035 < 0.05). The mentioned interactions were also confirmed by logistic regression, which showed that the effect of the *TP53* rs1042522 variant was modified by *MDM2* rs2279744. Table 3 illustrates the results of logistic regression. In subjects with a combination of the homozygous genotypes with the variant allele of *MDM2* rs2279744 and *TP53* rs1042522 (GG + Pro/Pro) the odds of AML was significantly increased (OR = 5.64) compared to those with wildtype and heterozygous genotype of *MDM2* rs2279744 and homozygous genotype with the variant allele of *TP53* rs1042522 (OR = 1.67). Regarding *MDM4* rs4245739 and *MDM2* rs3730485 interaction, logistic regression showed that in subjects with combined homozygous genotypes with the variant allele of *MDM4* rs4245739 and *MDM2* rs3730485 (AA + DD), the odds of AML was significantly decreased compared to those with wildtype and heterozygous genotype of *MDM4* rs4245739 and homozygous genotype with the variant allele of *MDM2* rs3730485. 

In Table 4, we illustrate the results of model-based multifactor dimensionality reduction (MB-MDR) analysis used to identify the higher-order interactions between the mentioned genetic variants and their association with odds of AML.

The synergistic effect and antagonism effect of on odds of AML are illustrated in Table 4.

The MB-MDR analysis suggested that that the four-locus model involving *MDM4* rs4245739, *TP53* rs1042522, *MDM2* rs2279744, and *MDM2* rs3730485 was significantly associated with increased odds of AML (*p* = 0.004) while the three-locus models involving *MDM4* rs4245739, *TP53* rs1042522, and *MDM2* rs2279744 were significantly associated with a decreased odds of AML (*p* = 0.001). The results of the permutation test established the sensitivity of the findings obtained by MB-MDR analysis.

### 3.4. TP53 rs1042522, MDM2 rs2279744, rs3730485, and MDM4 rs4245739 Variants and Clinical Features of AML Patients

The associations between *TP53* rs1042522, *MDM2* rs2279744, rs3730485, and *MDM4* rs4245739 variants and the clinical features of AML patients are illustrated as Supplementary Materials (Supplementary Materials Tables S1–S4. Table S1: Associations between demographic and clinical features and *TP53* rs1042522 variant in codominant, dominant, and recessive models, Table S2: Associations between demographic and clinical features and *MDM2* rs2279744 variant in codominant, dominant, and recessive models, Table S3: Associations between demographic and clinical features and *MDM2* rs3730485 variant in codominant, dominant, and recessive models, Table S4: Associations between demographic and clinical features and *MDM4* rs4245739 variant in codominant, dominant, and recessive models).

Significant associations were found between *TP53* rs1042522 and ELN risk, cytogenetic risk, *NPM1* mutation, and platelet (PLT) count. The post-hoc pairwise analysis performed after a significant association was found, revealed that high ELN risk, high cytogenetic risk, and homozygous variant genotype of *TP53* rs1042522 (adjusted p_FDR_ = 0.0345, respectively p_FDR_ = 0.0016). Also, the same analysis showed an association between low PLT count (<50,000 cells/mm^3^) and heterozygous genotype of *TP53* rs1042522 (adjusted p_FDR_ = 0.0248). *MDM2* rs2279744 variant was associated with AML type and PLT count. Post-hoc analysis also revealed that sAML was associated with wildtype and heterozygous (TT and TG) genotypes of *MDM2* rs2279744 (adjusted p_FDR_ = 0.0107) and PLT count (≥50,000 cells/mm^3^) was associated with homozygous genotype of the variant allele (GG) of *MDM2* rs2279744 (adjusted p_FDR_ = 0.00407). There was no statistical evidence for an association between *TP53* rs1042522 and *MDM2* rs2279744 variants and changes in white blood cells (WBC) count, Hemoglobin level, Hematocrit level, blasts percentage, lactate dehydrogenase (LDH) level, Eastern Cooperative Oncology Group (ECOG) performance status, treatment response, or treatment toxicity (*p* > 0.05). In the dominant model, the variant genotypes (II + DD) of *MDM2* rs3730485 were significantly associated with treatment toxicity (*p* = 0.019). Other significant associations for the *MDM2* rs3730485 variant were not noticed. Regarding the *MDM4* rs4245739 variant, there were significant associations with age at the time of diagnosis, Hemoglobin, and Hematocrit level (*p* < 0.05). The post-hoc pairwise analysis revealed that wildtype (AA) and heterozygous (AC) genotypes of *MDM4* rs4245739 were associated with older age at diagnosis (≥60 years) of AML patients (adjusted p_FDR_ = 0.0780) and the homozygous genotype with the variant allele (AA) was associated with Hemoglobin level ≥ 10 (adjusted p_FDR_ = 0.0303). Other significant associations between the *MDM4* rs4245739 variant and the abovementioned clinical features were not observed.

## 4. Discussion

*TP53* gene had an essential role in carcinogenesis and tumor progression being mutated in approximately half of human tumors. Several genetic abnormalities directly can influence the *TP53* activity and p53 functions. Alternatively, *TP53* activity and p53 functions may be suppressed by negative regulators such as MDM2 and MDM4 proteins (codified by *MDM2*, respectively *MDM4* gene) [6].

*TP53* rs1042522 (*TP53* Arg72Pro) was reported to affect the gene and protein function [7,8] due to the fact that the Pro amino acid of p53 protein is weaker for apoptosis induction and also for suppressing cellular transformation compared to Arg amino acid [1]. *MDM2* rs2279744 (*MDM2* 309T>G) and rs3730485 (*MDM2* del1518) and *MDM4* rs4245739 (*MDM4* 34091 C>A) variants were reported to influence the genes activity being associated with carcinogenesis and tumor progression [17,18,19,20]. Moreover, the interaction between *TP53* rs1042522 and *MDM2* rs2279744 [11,13,29] and between *MDM2* rs2279744 and rs3730485 [21] was reported. Therefore, our study evaluated the associations between the mentioned variants and odds of developing AML and the interactions of investigated *TP53*, *MDM2*, and *MDM4* variants and their association with odds of AML.

Our results showed that the variant genotypes of *TP53* rs1042522 are associated with higher odds of AML. Our results regarding AML susceptibility are similar to those reported recently by Bezerra MF et al. and Dunna NR et al. [11,16]. Studies included in a meta-analysis by Tian X et al. [7], investigating the *TP53* rs1042522 on AML patients [1,12,13,14,15], did not find associations between the variant genotypes of *TP53* rs1042522 and AML susceptibility, except the study performed by Dunna NR et al. [16] on 141 Japanese AML patients. The contradictory results may be explained by the different ethnicity of the investigated patients (Asian, Caucasian, Brazilian) or by the small number of AML patients included. However, to the best of our knowledge, our study reports the results of the largest cohort of AML patients. Moreover, our study focused on clinical features as well as somatic mutations; and associations between the homozygous variant genotype of *TP53* rs1042522 and high cytogenetic and ELN risk were also noticed. In turn, cytogenetic and ELN risk stratification scores were correlated with the outcome of AML patients [41]. An association between *NPM1* mutation (which is included in ELN 2017 risk stratification) and *TP53* rs1042522 was also noticed. Other associations between the investigated variants and genes mutations were not found. The study performed by Bezerra MF et al. [11] on 189 Brazilian AML patients investigated the molecular and cytogenetic risk scores according to *TP53* rs1042522, but they did not find any associations. The role of *TP53* rs1042522 in cancer susceptibility and progression may be explained by the fact that the p53 protein codified by the *TP53* gene with wild type genotype of rs1042522, interacts more efficiently with the MDM2 protein and in consequence the apoptosis is more efficient [42]. As a consequence, (theoretically) the variant genotypes of *TP53* rs1042522 might be risk factors for cancer development and/or progression. Part of published studies, such as the study performed by Furuya T et al. [42] found associations with cancer risk and progression of cancer, while others such as our study just for susceptibility.

*MDM4* rs4245739 was intensively studied in different types of cancer [18,22,35,36,43,44,45]. Part of them [35,36] found no association of this variant with cancer susceptibility, while others [22,43,44,45] found an association with reduced risk of cancer, mainly of breast cancer but not only. The published results are contradictory, depending on the cancer type. For example, the study performed by Stegeman S. et al. [18] reported an association of the *MDM4* rs4245739 A allele with an increased risk for prostate cancer [18]. On the other hand, Gonsmo L et al. [46] performed a study on ovarian and endometrial cancers, showing that the *MDM4* rs4245739 C allele represents a risk factor only for ovarian cancer [46]. According to our knowledge, none of the published studies included leukemic patients. Our study used the latest version for allele description based on NCBI’s and Ensemble’s genome browser and we found a significant association of the variant genotypes (AC and AA) with odds of AML, the results being similar to those reported for prostate cancer by Stegeman S et al. [18]. In previously published studies, the alleles were opposite annotated (A > C). Currently, even if A allele of *MDM4* rs4245739 is the ancestral allele, the C allele is considered as reference (C > A). Thus, we are unable to consider our results contradictory to those who reported associations of the variant genotypes of *MDM4* rs4245739 with a reduced risk of cancer.

In our study, *MDM2* rs2279744 and rs3730485 variants were not associated with AML susceptibility. Significant associations were noticed between *MDM2* rs2279744 variant and sAML, high PLT count, as well as between *MDM2* rs3730485 variant and treatment toxicity.

Contrary to our results regarding the AML susceptibility, the homozygous genotype with the variant allele of *MDM2* rs2279744 was reported as risk factor for AML in a cohort of 231 Chinese patients [1]. In a study performed on 575 pediatric AML patients of different races, significant association between the homozygous genotype with the variant allele of *MDM2* rs2279744 and AML susceptibility was noticed for Black and Hispanic races [25]. Similar results were obtained in the meta-analysis performed by He X et al. [47] where leukemia patients were included (including AML patients). Similar to our results regarding AML susceptibility, Falk I et al. [26] reported no association between *MDM2* rs2279744 variant and AML risk on a cohort of 189 Swedish patients, but association between the variant genotypes and low OS was observed. They suggested that *MDM2* rs2279744 variant and *TP53* mutational status might be used for prognostication, risk stratification, and treatment choice [26]. The associations between the investigated *MDM2* variants and cancer susceptibility and progression have a scientific substrate, being well known that these variants increase the *MDM2* expression and attenuate the *TP53* suppressor pathway. Recently, in other types of cancer, the variant genotypes of *MDM2* rs2279744 and/or rs3730485 were reported as risk factors for breast cancer but with a trend towards a good prognosis [48], for laryngeal [49], gynecological cancers [50], and in haplotype analysis for papillary thyroid carcinoma [17].

As we mentioned, the investigated variants influence the gene activity and the investigated genes may interact. In consequence, we performed an interaction analysis for the investigated variants. Our study demonstrated that *MDM2 rs2279744* interacts with the *TP53* rs1042522 variant and *MDM4* rs4245739 with the *MDM2* rs3730485 variant. Moreover, the mentioned variant interactions were associated with odds of AML. The homozygous genotype with the variant allele of *TP53* rs1042522 was associated with AML susceptibility, but combined with the homozygous genotype with the variant allele of *MDM2* rs2279744 increased the odds for AML 5.64 times. The variant allele of *MDM2* rs2279744 enhances the risk effect of the variant allele of *TP53* rs1042522. *MDM2 rs2279744* and *TP53* rs1042522 interaction and their association with the risk for cancer were recently reported by Cabezas M et al. [29] in therapy-related myeloid neoplasms. Our results are explained by the fact that *TP53* rs1042522 variant allele was reported to modify the p53 function and interaction with MDM2 protein [42], thus being reported by several studies as a risk factor for cancer. While, separately *MDM2* rs2279744 variant allele was reported to modify the MDM2 protein function [51] also being reported by several studies as a risk factor for cancer. Our study demonstrated their combined effect to the odds for AML.

In our study, the variant genotypes of *MDM4* rs4245739 were also found to be associated with AML susceptibility. Moreover, the combination of homozygous genotypes with the variant alleles of *MDM4* rs4245739 and *MDM2* rs3730485 decreased the odds of AML, while the combination of wildtype or heterozygous genotype of *MDM4* rs4245739 with homozygous genotype with the variant allele of *MDM2* rs3730485 increased the odds of AML. Our results suggesting that in subjects with homozygous genotype with the variant allele of *MDM2* rs3730485 the risk effect of the variant genotypes of *MDM4* rs4245739 is inverted (reversed effect).

Regarding OS, the investigated *TP53* rs1042522, *MDM2* rs2279744, rs3730485, and *MDM4* rs4245739 variants were not significantly associated with OS (*p* > 0.05) in either of the genetic models. Clinical characteristics such as treatment (low dose treatment), patients’ outcome, high LDH level, high PLT count, high WBC count, high cytogenetic and ELN risk, high age (>60 years) at diagnosis, and *FLT3* ITD mutation were associated with lower OS (*p* < 0.01), as expected. Part of the mentioned OS associations have been previously reported in the literature [2,41,52,53] and our group also reported part of these clinical associations on smaller cohorts of AML patients [31,32,53].

**Figure 1:**
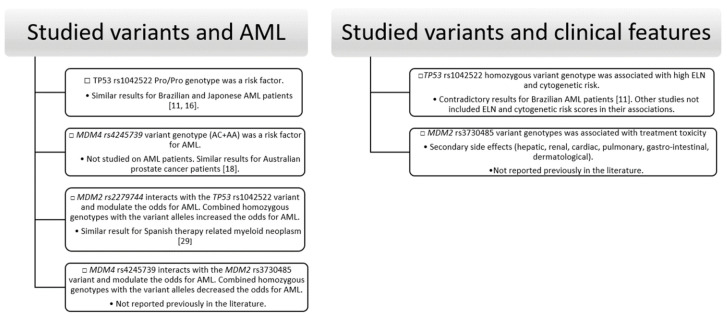
Summary diagram of significant results of the association analysis.

Briefly, our study showed association between *TP53* rs1042522 and *MDM4* rs4245739 variants and AML susceptibility, between *TP53* rs1042522 and PLT count, *NPM1* mutations (type A-D insertion), ELN, and cytogenetic risk. *MDM4* rs4245739 variant was also associated with age at diagnosis, and changes in Hemoglobin and Hematocrit level. *MDM2* rs2279744 variant was associated with secondary AML type and changes in PLT count and *MDM2* rs3730485 with secondary (hepatic, renal, cardiac, pulmonary, gastro-intestinal, dermatological) events as a result of treatment toxicity. MB-MDR framework and logistic regression demonstrated the interaction between *MDM2 rs2279744* and *TP53* rs1042522 variants and between *MDM4* rs4245739 and *MDM2* rs3730485 variants and also their association with AML susceptibility (Figure 1).

The novelty of our study consists of the simultaneous analysis of the four variants on a large cohort of adult AML patients. Also, according to our best knowledge, the present study is the first one to report the association between high ELN and cytogenetic risk scores and the *TP53* rs1042522 variant. Moreover, even if *MDM2* rs3730485 and *MDM4* rs4245739 were studied in several types of cancer, none of them included AML patients. Our study also focused on identifying the interactions of the mentioned variants and their association with odds of AML. However, our study has several limitations as well. One limitation is the lack of gene expression and protein level analysis. In addition, although the statistical models containing higher-order interactions between studied variant were internally validated using permutation samples, more studies with larger number of subjects would be needed to validate our associations. It is important to notice that *MDM4* rs4245739 variant was not in HWE in controls and AML group and once again this may be a demographic characteristic (considering that some unraveled modifying factors, at gene and environmental level, may be responsible). However, our frequencies of all investigates variants were similar to the allele frequencies reported by Ensembl Genome browser. Regarding other Romanian studies, similar frequencies of *TP53* rs1042522 alleles were found in a case-control study where Romanian colorectal cancer patients were included [54]. Another case-control study, where Romanian and German cholangiocarcinoma patients were included, investigated *TP53* rs1042522 (*TP53* Arg72Pro) and *MDM2* rs2279744 (*MDM2* 309T>G) and similar genotypes frequencies were reported [55].

## 5. Conclusions

Our findings provide evidence regarding the association between *TP53* rs1042522, *MDM4* rs4245739 variants, and AML susceptibility. A significant effect of interaction was found between *MDM2* rs2279744 and *TP53* rs1042522 variants and between *MDM4* rs4245739 and *MDM2* rs3730485 variants. The results of pairwise interactions showed that the effect of the *TP53* rs1042522 variant was modified by *MDM2* rs2279744, and patients with combined variant homozygous genotypes for *MDM2* rs2279744 and *TP53* rs1042522 have increased odds of AML. The results of MB-MDR analysis revealed significant higher-order interactions between the *TP53* rs1042522, *MDM2* rs2279744, rs3730485, and *MDM4* rs4245739 variants. The variant genotypes of *TP53* rs1042522 were significantly associated with adverse molecular and cytogenetic risk scores and also with *NPM1* mutation in AML patients.

## Figures and Tables

**Table 1:** TP53 rs1042522, MDM2 rs2279744, rs3730485, and MDM4 rs4245739 genotypes distribution.

Variants	Models	Genotypes	Controls, n_1_ (%)	AML Cases, n_2_ (%)	Crude Analysis			Adjusted by Age Group and Gender		
					OR, 95% IC	*p*-Value ^a^	Adjusted p_FDR_^b^	OR, 95% IC	*p*-Value ^a^	Adjusted p_FDR_^b^
*TP53* rs1042522	Codominant	Arg/Arg	217 (53.4)	225 (55.8)	Reference	**0.0003**	**0.0012**	Reference	**0.0005**	**0.0021**
		Arg/Pro	152 (37.4)	110 (27.3)	0.70 (0.51–0.95)			0.70 (0.52–0.96)		
		Pro/Pro	37 (9.1)	68 (16.9)	**1.77 (1.14–2.76)**			**1.74 (1.12–2.71)**		
	Dominant	Arg/Arg	217 (53.4)	225 (55.8)	Reference	0.4960	0.4960	Reference	0.5050	0.5050
		Arg/Pro + Pro/Pro	189 (46.6)	178 (44.2)	0.91 (0.69–1.20)			0.91 (0.69–1.20)		
	Recessive	Arg/Arg + Arg/Pro	369 (90.9)	335 (83.1)	Reference	**0.0009**	**0.0019**	Reference	**0.0014**	**0.0029**
		Pro/Pro	37 (9.1)	68 (16.9)	**2.02 (1.32–3.1)**			**1.98 (1.29–3.04)**		
	Overdominant	Arg/Arg + Pro/Pro	254 (62.6)	293 (72.7)	Reference	**0.0020**	**0.0027**	Reference	**0.0028**	**0.0037**
		Arg/Pro	152 (37.4)	110 (27.3)	0.63 (0.47–0.84)			0.63 (0.47–0.86)		
*MDM2* rs2279744	Codominant	TT	141 (34.7)	135 (33.5)	Reference	0.3464	0.4619	Reference	0.4192	0.5589
		TG	197 (48.5)	213 (52.9)	1.13 (0.83–1.53)			1.14 (0.84–1.15)		
		GG	68 (16.7)	55 (13.6)	0.84 (0.55–1.29)			0.89 (0.58–1.37)		
	Dominant	TT	141 (34.7)	135 (33.5)	Reference	0.7121	0.7121	Referemce	0.6093	0.6093
		TG + GG	265 (65.3)	268 (66.5)	1.06 (0.79–1.41)			1.08 (0.81–1.45)		
	Recessive	TT + TG	338 (83.3)	348 (86.4)	Reference	0.2190	0.4380	Reference	0.3156	0.5589
		GG	68 (16.7)	55 (13.6)	0.79 (0.53–1.16)			0.82 (0.56–1.21)		
	Overdominant	TT + GG	209 (51.5)	190 (47.1)	Reference	0.2178	0.4380	Reference	0.2290	0.5589
		TG	197 (48.5)	213 (52.9)	1.19 (0.90–1.57)			1.19 (0.90–1.57)		
*MDM2* rs3730485	Codominant	II	179 (44.1)	168 (41.7)	Reference	0.4373	0.4902	Reference	0.5066	0.6005
		ID	169 (41.6)	185 (45.9)	1.17 (0.87–1.57)			1.14 (0.84–1.53)		
		DD	58 (14.3)	50 (12.4)	0.92 (0.6–1.42)			0.90 (0.58–1.40)		
	Dominant	II	179 (44.1)	168 (41.7)	Reference	0.4902	0.4902	Reference	0.6005	0.6005
		ID + DD	227 (55.9)	235 (58.3)	1.10 (0.83–1.46)			1.08 (0.81–1.43)		
	Recessive	II + ID	348 (85.7)	353 (87.6)	Reference	0.4319	0.4902	Reference	0.4234	0.6005
		DD	58 (4.3)	50 (12.4)	0.85 (0.57–1.28)			0.85 (0.56–1.27)		
	Overdominant	II + DD	237 (58.4)	218 (54.1)	Reference	0.2198	0.4902	Reference	0.2834	0.6005
		ID	169 (41.6)	185 (45.9)	1.19 (0.90–1.57)			1.17 (0.88–1.57)		
*MDM4* rs4245739	Codominant	CC	83 (20.4)	57 (14.1)	Reference	**0.0145**	**0.0259**	Reference	**0.0159**	**0.0318**
		AC	114 (28.1)	144 (35.7)	**1.84 (1.21–2.79)**			**1.84 (1.21–2.80)**		
		AA	209 (51.5)	202 (50.1)	1.41 (0.95–2.08)			1.44 (0.97–2.13)		
	Dominant	CC	83 (20.4)	57 (14.1)	Reference	**0.0175**	0.0259	Reference	**0.0146**	**0.0318**
		AC + AA	323 (76.9)	346 (85.9)	**1.56 (1.08–2.26)**			**1.59 (1.09–2.30)**		
	Recessive	CC + AC	197 (48.5)	201 (49.9)	Reference	0.70	0.7001	Reference	0.827	0.8275
		AA	209 (51.5)	202 (50.1)	0.95 (0.72–1.25)			0.97 (0.73–1.28)		
	Overdominant	CC + AA	292 (71.9)	259 (64.3)	Reference	**0.0194**	**0.0259**	Reference	**0.0270**	**0.0360**
		AC	114 (28.1)	144 (35.7)	**1.42 (1.06–1.92)**			**1.40 (1.04–1.89)**		

Note: n_1_ = number of controls; n_2_ = number of AML cases; OR = odds ratio; 95%CI = 95% confidence interval; ^a^ *p*-values obtained from generalized linear models (GLMs) with a binomial distribution (logit-link); ^b^ *p*-values adjusted for multiple testing using the false discovery rate (FDR) with Benjamini and Hochberg procedure (n = 4 inheritance patterns); statistically significant results (*p* < 0.05) are highlighted in bold.

**Table 2:** Epistatic pairwise interactions between single nucleotide polymorphisms (SNPs) in odds of acute myeloid leukemia (AML).

*Studied Gene Polymorphisms*	Genetic Models	*MDM2* rs2279744	*TP53* rs1042522	*MDM4* rs4245739	*MDM2* rs3730485
*MDM2 rs2279744*	Codominant	0.412	0.183	0.587	0.197
	Dominant	0.610	0.488	0.296	0.139
	Overdominant	0.230	0.297	0.418	0.539
	Recessive	0.317	**0.044**	0.945	0.360
*TP53 rs1042522*	Codominant	0.428	**0.001**	0.181	0.580
	Dominant	0.609	0.506	0.551	0.714
	Overdominant	0.253	**0.003**	0.192	0.336
	Recessive	0.276	**0.002**	0.651	0.325
*MDM4 rs4245739*	Codominant	0.368	0.016	**0.016**	0.579
	Dominant	0.612	0.514	**0.015**	0.486
	Overdominant	0.229	0.030	**0.027**	0.999
	Recessive	0.847	0.813	0.830	**0.035**
*MDM2 rs3730485*	Codominant	0.701	0.506	0.383	0.508
	Dominant	0.480	0.567	0.423	0.602
	Overdominant	0.399	0.245	0.265	0.285
	Recessive	0.332	0.423	0.832	0.425

Note: Epistatic pairwise variants interactions were evaluated using SNPassoc package for R. The elements of the upper part of the matrix represent the *p*-values for epistatic pairwise interactions evaluated using the log-likelihood ratio test (LRT). The diagonal of the matrix contains the *p*-values obtained from LRT for the unadjusted (crude effect) of each variant. The elements of lower part of the matrix represents the *p*-values from LRT comparing the likelihood of the model containing the two variants and the best model containing a single variant. All the *p*-values were adjusted for age group (≥60 years) and gender. Statistically significant results (*p* < 0.05) are highlighted in bold.

**Table 3:** Logistic regression results with the main effects and gene–gene interaction terms according to the recessive genetic model.

	β (SE)	OR (95% CI)	*p*-Value
Main effects			
*MDM2* rs2279744 (GG vs. TG + TT)	−0.41(0.22)	0.67 (0.43–1.02)	0.064
*TP53* rs1042522 (Pro/Pro vs. Arg/Pro + Arg/Arg)	0.51 (0.24)	1.67 (1.05–2.69)	0.032
*MDM4* rs4245739 (AA vs. AC + CC)	0.10 (0.15)	1.10 (0.82–1.49)	0.521
*MDM2* rs3730485 (DD vs. ID + II)	026 (0.30)	1.30 (0.72–2.39)	0.386
Interaction effects			
*MDM2* rs2279744 and *TP53* rs1042522	1.22 (0.65)	3.38 (1.01–13.57)	0.050
*MDM4* rs4245739 and *MDM2* rs3730485	−0.92 (0.43)	0.40 (0.17–0.91)	0.031

Note: β = beta regression coefficients; SE = standard error, OR = odds ratio; CI = confidence interval; vs. = versus; *p*-value obtained from logistic model adjusted for age group (≥60 years) and gender.

**Table 4:** Results of the model-based multifactor dimensionality reduction method in the second-step analysis.

	Synergistic Effect					Antagonism Effect					Permutation Test
Interaction Models ^a^	N_H_^b^	Genotypes	β_H_^c^	W_H_^d^	p_H_^e^	N_L_^f^	Genotypes	β_L_^g^	W_L_^h^	p_L_^i^	Corrected *p*-Value
Two-order interaction models											
*MDM4* rs4245739+ *TP53* rs1042522	0	Na	Na	Na	Na	1	CC + ProPro	−0.89	3.09	0.078	0.100
*MDM2* rs3730485+ *MDM2* rs2279744	1	DD + TG	0.94	2.97	0.085	0	Na	Na	Na	Na	0.098
Three-order interaction models											
*MDM4* rs4245739+ *TP53* rs1042522+ *MDM2* rs2279744	1	AA + ProPro + GG	1.78	2.80	0.094	2	AC + ArgPro + TG CC + ProPro + TG	−1.14	10.70	**0.001**	**0.029**
*MDM2* rs3730485+ *MDM4* rs4245739+ *TP53* rs1042522	1	ID + AA + ArgPro	0.52	3.49	0.062	2	ID + CC + ProPro DD + AA + ProPro	−1.41	7.15	**0.008**	0.125
*MDM2* rs3730485+ *MDM4* rs4245739+ *MDM2* rs2279744	0	Na	Na	Na	Na	1	DD + ProPro + TT	−0.55	2.85	0.092	0.470
*MDM2* rs3730485+ *TP53* rs1042522+ *MDM2* rs2279744	1	II + ProPro + GG	1.08	2.75	0.097	0	Na	Na	Na	Na	0.495
Four-order interaction models											
*MDM4* rs4245739+ *TP53* rs1042522+ *MDM2* rs2279744+ *MDM2* rs3730485	2	CC + ArgPro + TG + II AC + ProPro + TG + II	1.39	8.39	**0.004**	4	AA + ProPro + GG + II AC + ArgArg + GG + ID CC + ProPro + TG + DD CC + ArgPro + TG + ID	−1.73	16.79	**0.00004**	**0.006**

Note. ^a^ Models obtained by the model-based multifactor dimensionality reduction method (MB-MDR); synergistic Effect (beta > 0) denoted that combination of genotypes has a positive impact on AML risk while antagonism effect (beta < 0) denoted a protective impact on AML risk. Na = not available; ^b^ number of genotypes assigned to the high-risk group; ^c^ beta regression coefficients for the high-risk group; ^d^ *p*-value for the high-risk group with adjustment for main effects of all studied SNPs and covariates: age category (>60 years) and sex; ^e^ Wald statistics for high-risk group; ^f^ number of genotypes assigned to the low-risk group; ^g^ beta regression coefficients for the low-risk group; ^h^ Wald statistics for low-risk group; ^i^ *p*-value for the low-risk group with adjustment for main effects of all studied SNPs and covariates: age category (>60 years) and sex; corrected *p*-value was permutation *p*-value. Statistically significant results (*p* < 0.05) are highlighted in bold.

## Supplementary Materials

The following are available online at https://www.mdpi.com/2077-0383/9/6/1672/s1, Table S1: Associations between demographic and clinical features and *TP53* rs1042522 variant in codominant, dominant and recessive models, Table S2: Associations between demographic and clinical features and *MDM2* rs2279744 variant in codominant, dominant and recessive models, Table S3: Associations between demographic and clinical features and *MDM2* rs3730485 variant in codominant, dominant and recessive models, Table S4: Associations between demographic and clinical features and *MDM4* rs4245739 variant in codominant, dominant and recessive models. The data used in the current study may be available upon reasonable request.
