# Supplementary material for: Association Analysis of TP53 rs1042522, MDM2 rs2279744, rs3730485, MDM4 rs4245739 Variants and Acute Myeloid Leukemia Susceptibility, Risk Stratification Scores, and Clinical Features: An Exploratory Study
Source: J Clin Med. 2020 Jun 1;9(6):1672. doi: 10.3390/jcm9061672 (PMC7355701; doi:10.3390/jcm9061672)
Supplement: Supplementary file 1 [file jcm-09-01672-s001.zip › Table S1_MI.docx]

Supplementary Table S1. Associations between demographic, clinical features and *TP53* rs1042522 variant in codominant, dominant and recessive genetic models

| Demographic and clinical factors | *TP53* rs1042522 Codominant model | | | | *TP53* rs1042522 Dominant model | | *TP53* rs1042522 Recessive model | |
| --- | --- | --- | --- | --- | --- | --- | --- | --- |
|  | Arg/Arg | Arg/Pro | Pro/Pro | p-value | Arg/Pro + Pro/Pro | p-value | Pro/Pro | p-value |
| Age categories, years |  |  |  |  |  |  |  |  |
| 18-39 | 43 (19.1%) | 21 (19.1%) | 6 (8.8%) | 0.1 | 27 (15.2%) | 0.123 | 6 (8.8%) | 0.113 |
| 40-59 | 67 (29.8%) | 43 (39.1%) | 27 (39.7%) |  | 70 (39.3%) |  | 27 (39.7%) |  |
| ≥60 | 115 (51.5%) | 46 (41.8%) | 35 (51.5%) |  | 81 (45.5%) |  | 35 (51.5%) |  |
| Gender |  |  |  |  |  |  |  |  |
| Female | 102 (45.3%) | 56 (50.9%) | 30 (44.1%) | 0.567 | 87 (48.3%) | 0.551 | 30 (44.1%) | 0.646 |
| Male | 123 (54.7%) | 54 (49.1%) | 38 (55.9%) |  | 92 (51.7%) |  | 38 (55.9%) |  |
| AML types |  |  |  |  |  |  |  |  |
| De novo AML | 171 (76%) | 91 (82.7%) | 54 (79.4%) | 0.43 | 145 (81.5%) | 0.244 | 54 (79.4%) | 0.947 |
| Secondary AML | 52 (23.1%) | 17 (15.5%) | 13 (19.1%) |  | 30 (16.9%) |  | 13 (19.1%) |  |
| Therapy-related AML | 2 (0.9%) | 2 (1.8%) | 1 (1.5%) |  | 3 (1.7%) |  | 1 (1.5%) |  |
| ELN 2017 risk |  |  |  |  |  |  |  |  |
| Favorable | 57 (25.3%) | 39 (35.8%) | 19 (27.9%) | 0.023* | 58 (32.8%) | 0.62 | 19 (27.9%) | 0.026* |
| Intermediate | 114 (50.7%) | 46 (42.2%) | 23 (33.8%) |  | 69 (39%) |  | 23 (33.8%) |  |
| Adverse | 54 (24%) | 24 (22%) | 26 (38.2%) |  | 50 (28.2%) |  | 26 (38.2%) |  |
| Cytogenetic risk |  |  |  |  |  |  |  |  |
| Favorable | 43 (19.8%) | 24 (22%) | 14 (20.9%) | 0.009** | 38 (21.6%) | 0.225 | 14 (20.9%) | 0.001** |
| Intermediate | 131 (60.4%) | 65 (59.6%) | 27 (40.3%) |  | 92 (52.3%) |  | 27 (40.3%) |  |
| Adverse | 43 (19.8%) | 20 (18.3%) | 26 (38.8%) |  | 46 (26.1%) |  | 26 (38.8%) |  |
| *FLT3* ITD mutation |  |  |  |  |  |  |  |  |
| Negative | 185 (82.2%) | 90 (81.8%) | 57 (93.8%) | 0.939 | 147 (82.6%) | 0.925 | 57 (83.8%) | 0.732 |
| Positive | 40 (17.8%) | 20 (18.2%) | 11 (16.2%) |  | 31 (17.4%) |  | 11 (16.2%) |  |
| *FLT3* D835 mutation |  |  |  |  |  |  |  |  |
| Negative | 214 (95.1%) | 104 (94.5%) | 63 (92.6%) | 0.736 | 167 (93.8%) | 0.571 | 63 (92.6%) | 0.394 |
| Positive | 11 (4.9%) | 6 (5.5%) | 5 (7.4%) |  | 11 (6.3%) |  | 5 (7.4%) |  |
| *FLT3* mutations |  |  |  |  |  |  |  |  |
| Negative | 177 (78.7%) | 84 (76.4%) | 55 (80.8%) | 0.769 | 139 (78.1%) | 0.889 | 55 (80.9%) | 0.587 |
| Positive | 48 (21.3%) | 26 (23.6%) | 13 (19.1%) |  | 39 (21.9%) |  | 13 (19.1%) |  |
| *NPM1* mutation |  |  |  |  |  |  |  |  |
| Negative | 190 (84.4%) | 87 (79.1%) | 51 (76.5%) | 0.0238* | 139 (78.1%) | 0.102 | 52 (76.5%) | 0.227 |
| Positive | 35 (15.6%) | 23 (20.9%) | 16 (23.5%) |  | 39 (21.9%) |  | 16 (23.5%) |  |
| *DNMT3A* mutation |  |  |  |  |  |  |  |  |
| Negative | 200 (88.9%) | 98 (89.1%) | 60 (88.2%) | 0.984 | 158 (88.8%) | 0.968 | 60 (88.2%) | 0.864 |
| Positive | 25 (11.1%) | 12 (10.9%) | 8 (11.8%) |  | 20 (11.2%) |  | 8 (11.8%) |  |
| WBC count |  |  |  |  |  |  |  |  |
| < 10000 cells/mm^3^ | 107 (47.6%) | 49 (44.5%) | 38 (55.9%) | 0.327 | 87 (48.9%) | 0.792 | 38 (55.9%) | 0.161 |
| ≥ 10000 cells/mm^3^ | 118 (52.4%) | 61 (55.5%) | 30 (44.1%) |  | 91 (51.1%) |  | 30 (44.1%) |  |
| Hemoglobil level |  |  |  |  |  |  |  |  |
| ≥ 10 g/dl | 63 (28%) | 40 (36.4%) | 14 (20.6%) | 0.069 | 54 (30.3%) | 0.608 | 14 (20.6%) | 0.092 |
| < 10 g/dl | 162 (72%) | 70 (63.6%) | 54 (79.4%) |  | 124 (69.7%) |  | 54 (79.4%) |  |
| Hematocrit level |  |  |  |  |  |  |  |  |
| < 26 | 107 (47.6%) | 56 (50.9%) | 38 (55.9% | 0.469 | 94 (52.8%) | 0.295 | 38 (55.9%) | 0.277 |
| ≥ 26 | 118 (52.4%) | 54 (49.1%) | 30 (44.1%) |  | 84 (47.2%) |  | 30 (44.1%) |  |
| Platelet count |  |  |  |  |  |  |  |  |
| < 50000 cells/mm^3^ | 103 (45.8%) | 68 (61.8%) | 40 (58.8%) | 0.011* | 108 (60.7%) | 0.003** | 40 (58.8%) | 0.242 |
| ≥ 50000 cells/mm^3^ | 122 (54.2%) | 42 (38.2%) | 28 (41.2%) |  | 70 (39.3%) |  | 28 (41.2%) |  |
| Blasts percentage |  |  |  |  |  |  |  |  |
| < 50% | 92 (40.9%) | 35 (31.8%) | 22 (32.4%) | 0.187 | 57 (32%) | 0.067 | 22 (32.4%) | 0.387 |
| ≥ 50% | 133 (59.1%) | 75 (68.2%) | 46 (67.6%) |  | 121 (68%) |  | 46 (67.6%) |  |
| LDH value |  |  |  |  |  |  |  |  |
| ≤ 600 IU/l | 92 (40.9%) | 45 (40.9%) | 31 (45.6%) | 0.774 | 76 (42.7%) | 0.715 | 31 (45.6%) | 0.474 |
| > 600 IU/l | 133 (59.1%) | 65 (59.1%) | 37 (54.4%) |  | 102 (57.3%) |  | 37 (54.4%) |  |
| ECOG score |  |  |  |  |  |  |  |  |
| ≤1 | 5 (2.2%) | 1 (0.9%) | 1 (1.5%) | 0.904 | 2 (1.1%) | 0.734 | 1 (1.5%) | 0.724 |
| 2 | 94 (41.8%) | 43 (39.1%) | 27 (39.7%) |  | 70 (39.3%) |  | 27 (39.7%) |  |
| 3 | 84 (37.3%) | 44 (40%) | 23 (33.8%) |  | 67 (37.6%) |  | 23 (33.8%) |  |
| 4 | 42 (18.7%) | 22 (20%) | 17 (25%) |  | 39 (21.9%) |  | 17 (25%) |  |
| Treatment |  |  |  |  |  |  |  |  |
| High dose | 108 (48%) | 57 (51.8%) | 37 (54.4%) | 0.216 | 94 (52.8%) | 0.087 | 37 (54.4%) | 0.716 |
| Low dose | 111 (49.3%) | 45 (40.9%) | 28 (41.2%) |  | 73 (41%) |  | 28 (41.2%) |  |
| High dose and Transplant | 6 (2.7%) | 8 (7.3%) | 3 (4.4%) |  | 11 (6.2%) |  | 3 (4.4%) |  |
| Response to treatment |  |  |  |  |  |  |  |  |
| Complete remission | 34 (15.1%0 | 26 (23.6%) | 10 (14.7%) | 0.340 | 36 (20.2%) | 0.387 | 10 (14.7%) | 0.534 |
| Partial remission | 48 (21.3%) | 15 (13.6%) | 14 (20.6%) |  | 29 (16.3%) |  | 14 (20.6%) |  |
| Resistance | 39 (17.4%) | 22 (20%) | 15 (22.1%) |  | 37 (20.8%) |  | 15 (22.1%) |  |
| Without response | 72 (32%) | 32 (29.1%) | 24 (35.3%) |  | 56 (31.5%) |  | 24 (35.3%) |  |
| Relapse | 32 (14.2%) | 15 (13.6%) | 5 (7.4%) |  | 20 (11.2%) |  | 5 (7.4%) |  |
| Toxicity |  |  |  |  |  |  |  |  |
| Absent | 101 (44.9%) | 41 (37.3%) | 32 (47.1%) | 0.325 | 73 (41%) | 0.435 | 32 (47.1%) | 0.478 |
| Positive | 124 (55.1%) | 69 (62.7%) | 36 (52.9%) |  | 105 (59%) |  | 36 (52.9%) |  |

Note. AML = Acute myeloid leukemia, ELN = European Leukemia Net 2017 risk stratification score, WBC = white blood cells, LDH = lactate dehydrogenase, ECOG = Eastern Cooperative Oncology Group performance status. Data were expressed as number and percentages; p-values were obtained by Chi-square or Fisher’s Exact test; statistical significance was reached if p-value < 0.05. p-value* <0.05, p-value**<0.005
