# Supplementary material for: Association Analysis of TP53 rs1042522, MDM2 rs2279744, rs3730485, MDM4 rs4245739 Variants and Acute Myeloid Leukemia Susceptibility, Risk Stratification Scores, and Clinical Features: An Exploratory Study
Source: J Clin Med. 2020 Jun 1;9(6):1672. doi: 10.3390/jcm9061672 (PMC7355701; doi:10.3390/jcm9061672)
Supplement: Supplementary file 1 [file jcm-09-01672-s001.zip › Table S2_MI.docx]

Supplementary Table S2. Associations between demographic and clinical features and *MDM2* rs2279744 variant in codominant, dominant and recessive genetic models

| Demographic and clinical factors | *MDM2* rs2279744 Codominant model | | | | *MDM2* rs2279744 Dominant model | | *MDM2* rs2279744 Recessive model | |
| --- | --- | --- | --- | --- | --- | --- | --- | --- |
|  | TT | TG | GG | p-value | TG + GG | p-value | GG | p-value |
| Age categories, years |  |  |  |  |  |  |  |  |
| 18-39 | 26 (19.3%) | 39 (18.3%) | 5 (9.1%) | 0.193 | 44 (16.4%) | 0.707 | 5 (9.1%) | 0.073 |
| 40-59 | 43 (31.9%) | 78 (36.6%) | 16 (29.1%) |  | 94 (35.1%) |  | 16 (29.1%) |  |
| ≥60 | 66 (48.9%) | 96 (45.1%) | 34 (61.8%) |  | 130 (48.5%) |  | 34 (61.8%) |  |
| Gender |  |  |  |  |  |  |  |  |
| Female | 58 (43%) | 100 (46.9%) | 30 (54.5%) | 0.346 | 130 (48.5%) | 0.292 | 30 (54.5%) | 0.207 |
| Male | 77 (57%) | 113 (53.1%) | 25 (45.5%) |  | 138 (51.5%) |  | 25 (45.5%) |  |
| AML types |  |  |  |  |  |  |  |  |
| De novo AML | 110 (81.5%) | 171 (80.3%) | 35 (63.6%) | 0.036* | 206 (76.9%) | 0.534 | 35 (63.6%) | 0.009* |
| Secondary AML | 23 (17%) | 39 (18.3%) | 20 (36.4%) |  | 59 (22%) |  | 20 (36.4%) |  |
| Therapy-related AML | 2 (1.5%) | 3 (1.4%) | 0 (0%) |  | 3 (1.1%) |  | 0 (0%) |  |
| ELN 2017 risk |  |  |  |  |  |  |  |  |
| Favorable | 40 (29.6%) | 59 (27.8%) | 16 (29.1%) | 0.983 | 75 (28.1%) | 0.873 | 16 (29.1%) | 0.918 |
| Intermediate | 59 (43.7%) | 98 (46.2 %) | 26 (47.3%) |  | 124 (46.45) |  | 26 (47.3%) |  |
| Adverse | 36 (26.7%) | 55 (25.9%) | 13 (23.6%) |  | 68 (25.5%) |  | 13 (23.6%) |  |
| Cytogenetic risk |  |  |  |  |  |  |  |  |
| Favorable | 30 (22.9%) | 39 (18.7%) | 12 (22.6%) | 0.865 | 51 (19.5%) | 0.713 | 12 (22.6%) | 0.825 |
| Intermediate | 73 (55.7%) | 122 (58.4%) | 28 (52.8%) |  | 150 (57.3%) |  | 28 (52.8%) |  |
| Adverse | 28 (21.4%) | 48 (23%) | 13 (24.5%) |  | 61 (23.3%) |  | 13 (24.5%) |  |
| *FLT3* ITD mutation |  |  |  |  |  |  |  |  |
| Negative | 112 (83%) | 176 (82.6%) | 44 (80%) | 0.88 | 220 (82.1%) | 0.828 | 44 (80%) | 0.618 |
| Positive | 23 (17%) | 37 (17.4%) | 11 (20%) |  | 48 (17.9%) |  | 11 (20%) |  |
| *FLT3* D835 mutation |  |  |  |  |  |  |  |  |
| Negative | 128 (94.8%) | 202 (94.8%) | 51 (92.7%) | 0.816 | 253 (94.4%) | 0.864 | 51 (92.7%) | 0.522 |
| Positive | 7 (5.2%) | 11 (5.2%) | 4 (7.3%) |  | 15 (5.6%) |  | 4 (7.3%) |  |
| *FLT3* mutations |  |  |  |  |  |  |  |  |
| Negative | 106 (78.5%) | 167 (78.4%) | 43 (78.2%) | 0.999 | 210 (78.4%) | 0.971 | 43 (78.2%) | 0.964 |
| Positive | 29 (21.5%) | 46 (21.6%) | 12 (21.85%) |  | 58 (21.6%) |  | 12 (21.8%) |  |
| *NPM1* mutation |  |  |  |  |  |  |  |  |
| Negative | 112 (83%) | 168 (78.9%) | 49 (89.1%) | 0.194 | 217 (81%) | 0.626 | 49 (89.1%) | 0.124 |
| Positive | 23 (17%) | 45 (21.1%) | 6 (10.9%) |  | 51 (19%) |  | 6 (10.9%) |  |
| *DNMT3A* mutation |  |  |  |  |  |  |  |  |
| Negative | 118 (87.4%) | 191 (89.7%) | 49 (89.1%) | 0.806 | 240 (89.6%) | 0.519 | 49 (89.1%) | 0.948 |
| Positive | 17 (12.6%) | 22 (10.3%) | 6 (10.9%) |  | 28 (10.4%) |  | 6 (10.9%) |  |
| WBC count |  |  |  |  |  |  |  |  |
| < 10000 cells/mm^3^ | 59 (43.7%) | 107 (50.2%) | 28 (50.9%) | 0.448 | 135 (50.4%) | 0.206 | 28 (50.9%) | 0.658 |
| ≥ 10000 cells/mm^3^ | 76 (56.3%) | 106 (49.8%) | 27 (49.1%) |  | 133 (49.6%) |  | 27 (49.1%) |  |
| Hemoglobil level |  |  |  |  |  |  |  |  |
| ≥ 10 g/dl | 35 (25.9%) | 61 (28.6%) | 21 (38.2%) | 0.237 | 82 (30.6%) | 0.33 | 21 (38.2%) | 0.108 |
| < 10 g/dl | 100 (74.1%) | 152 (71.4%) | 34 (61.8%) |  | 186 (69.4%) |  | 34 (61.8%) |  |
| Hematocrit level |  |  |  |  |  |  |  |  |
| < 26 | 73 (54.1%) | 102 (47.9%) | 26 (47.3%) | 0.487 | 128 (47.8%) | 0.232 | 26 (47.3%) | 0.678 |
| ≥ 26 | 62 (45.9%) | 111 (52.1%) | 29 (52.7%) |  | 140 (52.2%) |  | 29 (52.7%) |  |
| Platelet count |  |  |  |  |  |  |  |  |
| < 50000 cells/mm^3^ | 88 (65.2%) | 105 (49.3%) | 18 (32.7%) | <0.001*** | 123 (45.9%) | <0.001*** | 18 (32.7%) | 0.002** |
| ≥ 50000 cells/mm^3^ | 47 (34.8%) | 108 (50.7%) | 37 (67.3%) |  | 145 (54.1%) |  | 37 (67.3%) |  |
| Blasts percentage |  |  |  |  |  |  |  |  |
| < 50% | 49 (36.3%) | 78 (36.6%) | 22 (40%) | 0.881 | 100 (37.3%) | 0.842 | 22 (40%) | 0.617 |
| ≥ 50% | 86 (63.7%) | 135 (63.4%) | 33 (60%) |  | 168 (62.7%) |  | 33 (60%) |  |
| LDH value |  |  |  |  |  |  |  |  |
| ≤ 600 IU/l | 53 (39.3%) | 90 (42.3%) | 25 (45.5%) | 0.713 | 115 (42.9%) | 0.483 | 25 (45.5%) | 0.542 |
| > 600 IU/l | 82 (60.7%) | 123 (57.7%) | 30 (54.5%) |  | 153 (57.1%) |  | 30 (54.5%) |  |
| ECOG score |  |  |  |  |  |  |  |  |
| ≤1 | 2 (1.5%) | 5 (2.3%) | 0 (0%) | 0.054 | 5 (1.9%) | 0.344 | 0 (0%) | 0.068 |
| 2 | 56 (41.5%) | 93 (43.7%) | 15 (27.3%) |  | 108 (40.3%) |  | 15 (27.3%) |  |
| 3 | 44 (32.6%) | 83 (39%) | 24 (43.6%) |  | 107 (39.9%) |  | 24 (43.6%) |  |
| 4 | 33 (24.4%) | 32 (15%) | 16 (29.1%) |  | 48 (17.9%) |  | 16 (29.1%) |  |
| Treatment |  |  |  |  |  |  |  |  |
| High dose | 65 (48.1%) | 112 (52.6%) | 25 (45.5%) | 0.85 | 137 (51.1%) | 0.853 | 25 (45.5%) | 0.7 |
| Low dose | 64 (47.4%) | 92 (43.2%) | 28 (50.9%) |  | 120 (44.8%) |  | 28 (50.9%) |  |
| High dose and Transplant | 6 (4.4%) | 9 (4.2%) | 2 (3.6%) |  | 11 (4.1%) |  | 2 (3.6%) |  |
| Response to treatment |  |  |  |  |  |  |  |  |
| Complete remission | 26 (19.3%) | 38 (17.8%) | 6 (10.9%) | 0.497 | 44 (16.4%) | 0.243 | 6 (10.9%) | 0.583 |
| Partial remission | 20 (14.8%) | 44 (20.7%) | 13 (23.6%) |  | 57 (21.3%) |  | 13 (23.6%) |  |
| Resistance | 32 (23.7%) | 34 (16%) | 10 (18.2%) |  | 44 (16.4%) |  | 10 (18.2%) |  |
| Without response | 39 (28.9%) | 69 (32.4%) | 20 (36.4%) |  | 89 (33.2%) |  | 20 (36.4%) |  |
| Relapse | 18 (13.3%) | 28 (13.1%) | 6 (10.9%) |  | 34 (12.7%) |  | 6 (10.9%) |  |
| Toxicity |  |  |  |  |  |  |  |  |
| Absent | 54 (40%) | 91 (42.7%) | 29 (52.7%) | 0.27 | 120 (44.8%) | 0.361 | 29 (52.7%) | 0.124 |
| Positive | 81 (60%) | 122 (57.3%) | 26 (47.3%) |  | 148 (55.2%) |  | 26 (47.3%) |  |

Note. AML = Acute myeloid leukemia, ELN = European Leukemia Net 2017 risk stratification score, WBC = white blood cells, LDH = lactate dehydrogenase, ECOG = Eastern Cooperative Oncology Group performance status. Data were expressed as number and percentages; p-values were obtained by Chi-square or Fisher’s Exact test; statistical significance was reached if p-value < 0.05. p-value* <0.05, p-value**<0.005, p-value***<0.001
