# Supplementary material for: Association Analysis of TP53 rs1042522, MDM2 rs2279744, rs3730485, MDM4 rs4245739 Variants and Acute Myeloid Leukemia Susceptibility, Risk Stratification Scores, and Clinical Features: An Exploratory Study
Source: J Clin Med. 2020 Jun 1;9(6):1672. doi: 10.3390/jcm9061672 (PMC7355701; doi:10.3390/jcm9061672)
Supplement: Supplementary file 1 [file jcm-09-01672-s001.zip › Table S3_MI.docx]

Supplementary Table S3. Associations between demographic and clinical features and *MDM2* rs3730485 variant in codominant, dominant and recessive genetic models

| Demographic and clinical factors | *MDM2* rs3730485 Codominant model | | | | *MDM2* rs3730485 Dominant model | | *MDM2* rs3730485 Recessive model | |
| --- | --- | --- | --- | --- | --- | --- | --- | --- |
|  | II | ID | DD | p-value | ID + DD | p-value | DD | p-value |
| Age categories |  |  |  |  |  |  |  |  |
| 18-39 years | 23 (13.7%) | 37 (20%) | 10 (20%) | 0.317 | 47 (20%) | 0.183 | 10 (20%) | 0.618 |
| 40-59 years | 56 (33.3%) | 67 (36.2%) | 14 (28.0%) |  | 81 (34.5%) |  | 14 (28%) |  |
| ≥60 years | 89 (53%) | 81 (43.8%) | 26 (52%) |  | 107 (45.5%) |  | 26 (52%) |  |
| Gender |  |  |  |  |  |  |  |  |
| Female | 87 (51.8%) | 79 (42.7%) | 22 (44%) | 0.214 | 101 (43%) | 0.081 | 22 (44%) | 0.688 |
| Male | 81 (48.2%) | 106 (57.3%) | 28 (56%) |  | 134 (57%) |  | 28 (56%) |  |
| AML types |  |  |  |  |  |  |  |  |
| De novo AML | 124 (73.8%) | 149 (80.5%) | 43 (86%) | 0.363 | 192 (81.7%) | 0.129 | 43 (86%) | 0.381 |
| Secondary AML | 41 (24.4%) | 34 (18.4%) | 7 (14%) |  | 41 (17.4%) |  | 7 (14%) |  |
| Therapy-related AML | 3 (1.8%) | 2 (1.1%) | 0 (0%) |  | 2 (0.9%) |  | 0 (0%) |  |
| ELN 2017 risk |  |  |  |  |  |  |  |  |
| Favorable | 43 (25.6%) | 53 (28.8%) | 19 (28.6%) | 0.239 | 72 (30.8%) | 0.519 | 19 (38%) | 0.084 |
| Intermediate | 79 (47.0%) | 80 (43.5 %) | 24 (48%) |  | 104 (44.4) |  | 24 (48%) |  |
| Adverse | 46 (27.4%) | 51 (27.7%) | 7 (14%) |  | 58 (24.8%) |  | 7 (14%) |  |
| Cytogenetic risk |  |  |  |  |  |  |  |  |
| Favorable | 30 (18.3%) | 39 (21.7%) | 12 (24.5%) | 0.235 | 51 (22.3%) | 0.419 | 12 (24.5%) | 0.084 |
| Intermediate | 93 (56.7%) | 98 (54.4%) | 32 (65.3%) |  | 130 (56.8%) |  | 32 (65.3%) |  |
| Adverse | 41 (25%) | 43 (23.9%) | 5 (10.2%) |  | 48 (21%) |  | 5 (10.2%) |  |
| *FLT3* ITD mutation |  |  |  |  |  |  |  |  |
| Negative | 133 (79.2%) | 157 (84.9%) | 42 (84%) | 0.355 | 199 (84.7%) | 0.152 | 42 (84%) | 0.748 |
| Positive | 35 (20.8%) | 28 (15.1%) | 8 (16%) |  | 36 (15.3%) |  | 8 (16%) |  |
| *FLT3* D835 mutation |  |  |  |  |  |  |  |  |
| Negative | 157 (93.5%) | 177 (95.7%) | 47 (94%) | 0.645 | 224 (95.3%) | 0.416 | 47 (94%) | 0.745 |
| Positive | 11 (6.5%) | 8 (4.3%) | 3 (6%) |  | 11 (4.7%) |  | 3 (6%) |  |
| *FLT3* mutations |  |  |  |  |  |  |  |  |
| Negative | 127 (75.6%) | 150 (81.1%) | 39 (78%) | 0.456 | 189 (80.4%) | 0.245 | 39 (78%) | 0.94 |
| Positive | 41 (24.4%) | 35 (18.9%) | 11 (22%) |  | 46 (19.6%) |  | 11 (22%) |  |
| *NPM1* mutation |  |  |  |  |  |  |  |  |
| Negative | 142 (84.5%) | 150 (81.1%) | 37 (74%) | 0.233 | 187 (79.6%) | 0.206 | 37 (74%) | 0.136 |
| Positive | 26 (15.5%) | 35 (18.9%) | 13 (26%) |  | 48 (20.4%) |  | 13 (26%) |  |
| *DNMT3A* mutation |  |  |  |  |  |  |  |  |
| Negative | 154 (91.7%) | 163 (88.1%) | 41 (82%) | 0.149 | 204 (86.8%) | 0.127 | 41 (82%) | 0.101 |
| Positive | 14 (8.3%) | 22 (11.9%) | 9 (18%) |  | 31 (13.2%) |  | 9 (18%) |  |
| WBC count |  |  |  |  |  |  |  |  |
| < 10000 cells/mm^3^ | 84 (50%) | 90 (48.6%) | 20 (40%) | 0.454 | 110 (46.8%) | 0.527 | 20 (40%) | 0.218 |
| ≥ 10000 cells/mm^3^ | 84 (50%) | 95 (51.4%) | 30 (60%) |  | 125 (53.2%) |  | 30 (60%) |  |
| Hemoglobil level |  |  |  |  |  |  |  |  |
| ≥ 10 g/dl | 48 (28.6%) | 58 (31.4%) | 11 (22%) | 0.427 | 69 (29.4%) | 0.863 | 11 (22%) | 0.242 |
| < 10 g/dl | 120 (71.4%) | 127 (68.6%) | 39 (78%) |  | 166 (70.6%) |  | 39 (78%) |  |
| Hematocrit level |  |  |  |  |  |  |  |  |
| < 26 | 85 (50.6%) | 91 (49.2%) | 25 (50%) | 0.966 | 116 (49.4%) | 0.807 | 25 (50%) | 0.985 |
| ≥ 26 | 83 (49.4%) | 94 (50.8%) | 25 (50%) |  | 119 (50.6%) |  | 25 (50%) |  |
| Platelet count |  |  |  |  |  |  |  |  |
| < 50000 cells/mm^3^ | 81 (48.2%) | 102 (55.1%) | 28 (56%) | 0.369 | 130 (55.3%) | 0.159 | 28 (56%) | 0.582 |
| ≥ 50000 cells/mm^3^ | 87 (51.8%) | 83 (44.9%) | 22 (44%) |  | 105 (44.7%) |  | 22 (44%) |  |
| Blasts percentage |  |  |  |  |  |  |  |  |
| < 50% | 63 (37.5%) | 69 (37.5%) | 17 (34%) | 0.897 | 86 (36.6%) | 0.853 | 17 (34%) | 0.642 |
| ≥ 50% | 105 (62.5%) | 116 (62.7%) | 33 (66%) |  | 149 (63.4%) |  | 33 (66%) |  |
| LDH value |  |  |  |  |  |  |  |  |
| ≤ 600 IU/l | 67 (39.9%) | 81 (43.8%) | 20 (40%) | 0.734 | 101 (43%) | 0.534 | 20 (40%) | 0.796 |
| > 600 IU/l | 101 (60.1%) | 104 (56.2%) | 30 (60%) |  | 134 (57%) |  | 30 (60%) |  |
| ECOG score |  |  |  |  |  |  |  |  |
| ≤1 | 2 (1.2%) | 5 (2.7%) | 0 (0%) | 0.09 | 5 (2.1%) | 0.272 | 0 (0%) | 0.115 |
| 2 | 63 (37.5%) | 86 (46.5%) | 15 (30%) |  | 101 (43%) |  | 15 (30%) |  |
| 3 | 62 (36.9%) | 63 (34.1%) | 26 (52%) |  | 89 (37.9%) |  | 26 (52%) |  |
| 4 | 41 (24.4%) | 31 (16.8%) | 9 (18%) |  | 40 (17%) |  | 9 (18%) |  |
| Treatment |  |  |  |  |  |  |  |  |
| High dose | 87 (51.8%) | 94 (50.8%) | 21 (42%) | 0.449 | 115 (48.9%) | 0.291 | 21 (42%) | 0.431 |
| Low dose | 77 (45.8%) | 81 (43.8%) | 26 (52%) |  | 107 (45.5%) |  | 26 (52%) |  |
| High dose and Transplant | 4 (2.4%) | 10 (5.4%) | 3 (6%) |  | 13 (5.5%) |  | 3 (6%) |  |
| Response to treatment |  |  |  |  |  |  |  |  |
| Complete remission | 26 (15.5%) | 33 (17.8%) | 11 (22%) | 0.563 | 44 (18.7%) | 0.602 | 11 (22%) | 0.579 |
| Partial remission | 33 (19.6%) | 34 (18.4%) | 10 (20%) |  | 44 (18.7%) |  | 10 (20%) |  |
| Resistance | 37 (22%) | 29 (15.7%) | 10 (20%) |  | 39 (16.6%) |  | 10 (20%) |  |
| Without response | 53 (31.5%) | 59 (31.9%) | 16 (32%) |  | 75 (31.9%) |  | 16 (32%) |  |
| Relapse | 19 (11.3%) | 30 (16.2%) | 3 (6%) |  | 33 (14%) |  | 3 (6%) |  |
| Toxicity |  |  |  |  |  |  |  |  |
| Absent | 84 (50%) | 72 (38.9%) | 18 (36%) | 0.061 | 90 (38.3%) | 0.019* | 18 (36%) | 0.274 |
| Positive | 84 (50%) | 113 (61.1%) | 32 (64%) |  | 145 (61.7%) |  | 32 (64%) |  |

Note. AML = Acute myeloid leukemia, ELN = European Leukemia Net 2017 risk stratification score, WBC = white blood cells, LDH = lactate dehydrogenase, ECOG = Eastern Cooperative Oncology Group performance status. Data were expressed as number and percentages; p-values were obtained by Chi-square or Fisher’s Exact test; statistical significance was reached if p-value < 0.05. p-value* <0.05
