# Supplementary material for: Association Analysis of TP53 rs1042522, MDM2 rs2279744, rs3730485, MDM4 rs4245739 Variants and Acute Myeloid Leukemia Susceptibility, Risk Stratification Scores, and Clinical Features: An Exploratory Study
Source: J Clin Med. 2020 Jun 1;9(6):1672. doi: 10.3390/jcm9061672 (PMC7355701; doi:10.3390/jcm9061672)
Supplement: Supplementary file 1 [file jcm-09-01672-s001.zip › Table S4_MI.docx]

Supplementary Table S4. Associations between demographic and clinical features and *MDM4* rs4245739 variant in codominant, dominant and recessive genetic models

| Demographic and clinical factors | *MDM4* rs4245739 Codominant model | | | | *MDM4* rs4245739 Dominant model | | *MDM4* rs4245739 Recessive model | |
| --- | --- | --- | --- | --- | --- | --- | --- | --- |
|  | CC | AC | AA | p-value | AC + AA | p-value | AA | p-value |
| Age categories, years |  |  |  |  |  |  |  |  |
| 18-39 | 15 (26.3%) | 30 (20.8%) | 25 (12.4%) | 0.07 | 55 (15.9%) | 0.135 | 25 (12.4%) | 0.024* |
| 40-59 | 19 (33.3%) | 48 (33.3%) | 70 (34.7%) |  | 118 (34.1%) |  | 70 (34.7%) |  |
| ≥60 | 23 (40.4%) | 66 (45.8%) | 107 (53%) |  | 173 (50.0%) |  | 107 (53%) |  |
| Gender |  |  |  |  |  |  |  |  |
| Female | 25 (43.9%) | 65 (45.1%) | 98 (48.5%) | 0.744 | 163 (47.1%) | 0.649 | 98 (48.5%) | 0.452 |
| Male | 32 (56.1%) | 79 (54.9%) | 104 (51.5%) |  | 183 (52.9%) |  | 104 (51.5%) |  |
| AML types |  |  |  |  |  |  |  |  |
| De novo AML | 42 (73.7%) | 115 (79.9%) | 159 (78.7%) | 0.740 | 274 (79.2%) | 0.392 | 159 (78.7%) | 0.908 |
| Secondary AML | 15 (26.3%) | 27 (18.8%) | 40 (19.8%) |  | 67 (19.4%) |  | 40 (19.8%) |  |
| Therapy-related AML | 0 (0%) | 2 (1.4%) | 3 (1.5%) |  | 5 (1.4%) |  | 3 (1.5%) |  |
| ELN 2017 risk |  |  |  |  |  |  |  |  |
| Favorable | 18 (31.6%) | 46 (31.9%) | 51 (25.4%) | 0.467 | 97 (28.1%) | 0.865 | 51 (25.4%) | 0.186 |
| Intermediate | 25 (43.9%) | 67 (46.5 %) | 91 (45.3%) |  | 158 (45.8%) |  | 91 (45.3%) |  |
| Adverse | 14 (24.6%) | 31 (21.5%) | 59 (29.4%) |  | 90 (26.1%) |  | 59 (29.4%) |  |
| Cytogenetic risk |  |  |  |  |  |  |  |  |
| Favorable | 11 (20.4%) | 31 (22%) | 39 (19.7%) | 0.903 | 70 (20.6%) | 0.964 | 39 (19.7%) | 0.725 |
| Intermediate | 30 (55.6%) | 82 (58.2%) | 111 (56.1%) |  | 193 (56.9%) |  | 111 (56.1%) |  |
| Adverse | 13 (24.1%) | 28 (19.9%) | 48 (24.2%) |  | 76 (22.4%) |  | 48 (24.2%) |  |
| *FLT3* ITD mutation |  |  |  |  |  |  |  |  |
| Negative | 49 (86%) | 112 (84.7%) | 116 (79.7%) | 0.359 | 283 (81.8%) | 0.444 | 161 (79.7%) | 0.157 |
| Positive | 8 (14%) | 22 (15.3%) | 41 (20.3%) |  | 63 (18.2%) |  | 41 (20.3%) |  |
| *FLT3* D835 mutation |  |  |  |  |  |  |  |  |
| Negative | 55 (96.5%) | 133 (92.4%) | 193 (95.5%) | 0.343 | 326 (94.2%) | 0.753 | 193 (95.5%) | 0.374 |
| Positive | 2 (3.5%) | 11 (7.6%) | 9 (4.5%) |  | 20 (5.8%) |  | 9 (4.5%) |  |
| *FLT3* mutations |  |  |  |  |  |  |  |  |
| Negative | 47 (82.5%) | 113 (78.5%) | 156 (77.2%) | 0.698 | 269 (77.7%) | 0.423 | 156 (77.2%) | 0.562 |
| Positive | 10 (17.5%) | 31 (21.5%) | 46 (22.8%) |  | 77 (22.3%) |  | 46 (22.8%) |  |
| *NPM1* mutation |  |  |  |  |  |  |  |  |
| Negative | 43 (75.4%) | 122 (84.7%) | 164 (81.2%) | 0.301 | 286 (82.7%) | 0.192 | 164 (81.2%) | 0.815 |
| Positive | 14 (24.6%) | 22 (15.3%) | 38 (18.8%) |  | 60 (17.3%) |  | 38 (18.8%) |  |
| *DNMT3A* mutation |  |  |  |  |  |  |  |  |
| Negative | 49 (86%) | 126 (87.5%) | 183 (90.6%) | 0.506 | 309 (89.3%) | 0.458 | 183 (90.6%) | 0.261 |
| Positive | 8 (14%) | 18 (12.5%) | 19 (9.4%) |  | 37 (10.7%) |  | 19 (9.4%) |  |
| WBC count |  |  |  |  |  |  |  |  |
| < 10000 cells/mm^3^ | 26 (45.6%) | 64 (44.4%) | 104 (51.5%) | 0.399 | 168 (48.6%) | 0.681 | 104 (51.5%) | 0.178 |
| ≥ 10000 cells/mm^3^ | 31 (54.4%) | 80 (55.6%) | 98 (48.5%) |  | 178 (51.4%) |  | 98 (48.5%) |  |
| Hemoglobil level |  |  |  |  |  |  |  |  |
| ≥ 10 g/dl | 23 (40.4%) | 45 (31.3%) | 49 (24.3%) | 0.047* | 94 (27.2%) | 0.042* | 49 (24.3%) | 0.034* |
| < 10 g/dl | 34 (59.5%) | 99 (68.8%) | 153 (75.7%) |  | 252 (72.8%) |  | 153 (75.7%) |  |
| Hematocrit level |  |  |  |  |  |  |  |  |
| < 26 | 20 (35.1%) | 74 (51.4%) | 107 (53.%) | 0.053 | 181 (52.3%) | 0.016* | 107 (53%) | 0.213 |
| ≥ 26 | 37 (64.9%) | 70 (48.6%) | 47 (44%) |  | 165 (47.7%) |  | 95 (47%) |  |
| Platelet count |  |  |  |  |  |  |  |  |
| < 50000 cells/mm^3^ | 31 (54.4%) | 72 (50%) | 108 (53.5%) | 0.773 | 180 (52%) | 0.741 | 108 (53.5%) | 0.655 |
| ≥ 50000 cells/mm^3^ | 26 (45.6%) | 72 (50%) | 94 (46.5%) |  | 166 (48%) |  | 94 (46.5%) |  |
| Blasts percentage |  |  |  |  |  |  |  |  |
| < 50% | 20 (35.1%) | 60 (41.7%) | 69 (34.2%) | 0.344 | 129 (37.3%) | 0.750 | 69 (34.2%) | 0.241 |
| ≥ 50% | 37 (64.9%) | 84 (58.3%) | 133 (65.8%) |  | 217 (62.7%) |  | 133 (65.8%) |  |
| LDH value |  |  |  |  |  |  |  |  |
| ≤ 600 IU/l | 25 (43.9%) | 64 (44.4%) | 79 (39.1%) | 0.573 | 143 (41.3%) | 0.720 | 79 (39.1%) | 0.293 |
| > 600 IU/l | 32 (56.1%) | 80 (55.6%) | 123 (60.9%) |  | 203 (58.7%) |  | 123 (60.9%) |  |
| ECOG score |  |  |  |  |  |  |  |  |
| ≤1 | 2 (3.5%) | 4 (2.8%) | 1 (0.5%) | 0.572 | 5 (1.4%) | 0.652 | 1 (0.5%) | 0.313 |
| 2 | 22 (38.6%) | 58 (40.3%) | 84 (41.6%) |  | 142 (41%) |  | 84 (41.6%) |  |
| 3 | 23 (40.4%) | 53 (36.8%) | 75 (37.1%) |  | 128 (37%) |  | 75 (37.1%) |  |
| 4 | 10 (17.5%) | 29 (20.1%) | 42 (20.8%) |  | 71 (20.5%) |  | 42 (20.8%) |  |
| Treatment |  |  |  |  |  |  |  |  |
| High dose | 27 (47.4%) | 80 (55.6%) | 95 (47%) | 0.522 | 175 (50.6%) | 0.854 | 95 (47%) | 0.410 |
| Low dose | 27 (47.4%) | 60 (41.7%) | 97 (48%) |  | 157 (45.4%) |  | 97 (48%) |  |
| High dose and Transplant | 3 (5.3%) | 4 (2.8%) | 10 (5%) |  | 14 (4%) |  | 10 (5%) |  |
| Response to treatment |  |  |  |  |  |  |  |  |
| Complete remission | 9 (15.8%0 | 30 (20.8%) | 31 (15.3%) | 0.055 | 61 (17.6%) | 0.055 | 31 (15.3%) | 0.718 |
| Partial remission | 8 (14%) | 33 (22.9%) | 36 (17.8%) |  | 69 (19.9%) |  | 36 (17.8%) |  |
| Resistance | 12 (21.1%) | 25 (17.4%) | 39 (19.3%) |  | 64 (18.5%) |  | 39 (19.3%) |  |
| Without response | 14 (24.6%) | 46 (31.9%) | 68 (33.7%) |  | 114 (32.9%) |  | 68 (33.7%) |  |
| Relapse | 14 (24.6%) | 10 (6.9%) | 28 (13.9%) |  | 38 (11%) |  | 28 (13.9%) |  |
| Toxicity |  |  |  |  |  |  |  |  |
| Absent | 22 (38.6%) | 62 (43.1%) | 90 (44.6%) | 0.725 | 152 (43.9%) | 0.451 | 90 (44.6%) | 0.575 |
| Positive | 35 (61.4%) | 82 (56.9%) | 112 (55.4%) |  | 194 (56.1%) |  | 112 (55.4%) |  |

Note. AML = Acute myeloid leukemia. ELN = European Leukemia Net 2017 risk stratification score. WBC = white blood cells. LDH = lactate dehydrogenase. ECOG = Eastern Cooperative Oncology Group performance status. Data were expressed as number and percentages; p-values were obtained by Chi-square or Fisher’s Exact test; statistical significance was reached if p-value < 0.05. p-value* <0.05.
